# Sirtuin 1 and Aging Theory for Chronic Obstructive Pulmonary Disease

**DOI:** 10.1155/2015/897327

**Published:** 2015-07-05

**Authors:** V. Conti, G. Corbi, V. Manzo, G. Pelaia, A. Filippelli, A. Vatrella

**Affiliations:** ^1^Department of Medicine and Surgery, University of Salerno, 84081 Baronissi, Italy; ^2^Department of Medicine and Health Sciences, University of Molise, Via De Sanctis, 86100 Campobasso, Italy; ^3^Department of Medical and Surgical Sciences, University “Magna Graecia” of Catanzaro, Campus Universitario “S. Venuta”, Viale Europa-Località Germaneto, 88100 Catanzaro, Italy

## Abstract

Chronic Obstructive Pulmonary disease (COPD) is an inflammatory syndrome that represents an increasing health problem, especially in the elderly population. Drug therapies are symptomatic and inadequate to contrast disease progression and mortality. Thus, there is an urgent need to clarify the molecular mechanisms responsible for this condition in order to identify new biomarkers and therapeutic targets. Processes including oxidant/antioxidant, protease/antiprotease, and proliferative/antiproliferative balance and control of inflammatory response become dysfunctional during aging as well as in COPD. Recently it was suggested that Sirtuin 1 (SIRT1), an antiaging molecule involved in the response to oxidative stress and chronic inflammation, is implicated in both development and progression of COPD. The present review focuses on the involvement of SIRT1 in the regulation of redox state, inflammation, and premature senescence, all crucial characteristics of COPD phenotypes. Recent evidence corroborating the statement of the “aging theory for COPD” was also discussed.

## 1. Introduction

Chronic Obstructive Pulmonary disease (COPD) is a common respiratory disorder characterized by a progressive decline in lung function and chronic inflammatory response [1, 2]. The prevalence is particularly high in the elderly and it is expected to become the third leading cause of death worldwide by 2020 [2, 3]. Existing therapies are essentially symptomatic and not able to positively influence the course of the disease [4–6]. Moreover, the guidelines for respiratory disorders, including COPD, are not well clear and thus often disappointing [7, 8].

Nowadays it is evident that COPD represents a syndrome with several phenotypes differentiated by symptoms, number of exacerbations, and therapy response [6, 9]. The molecular mechanisms determining COPD are not still clarified because of its complexity and great heterogeneity. Therefore, it is necessary to better understand such mechanisms in order to identify novel biomarkers and more effective treatments.

Most of COPD patients are elderly, and many features of COPD are shared with aged lung. Recently, the statement of “the aging hypothesis for COPD” suggested that this syndrome represents a manifestation of accelerated aging [10]. Fundamental processes including oxidant/antioxidant, protease/antiprotease, and proliferative/antiproliferative balance and control of inflammatory response become dysfunctional during aging as well as in COPD [10, 11].

To confirm this relationship, the “MARK-AGE,” a large-scale study aiming to identify a valid set of aging biomarkers, considered several physical parameters as “classical” candidates, including lung function (as forced expiratory volume in 1 second [FEV1] and forced vital capacity [FVC]) together with immunological, systemic inflammation and oxidative stress markers [12].

Some molecules related to chronic inflammation and oxidative stress and associated with cellular senescence have been used to characterize the aging phenotype [13] and might characterize COPD [10].

More recently, it has been suggested that factors, now indicated as “antiaging molecules” such as histone/protein deacetylases HDACs and sirtuins, could be implicated in both development and progression of COPD [11, 14].

## 2. Oxidative Stress and COPD

Cigarette smoke (CS), the major risk factor for pulmonary diseases, is an exceptional source of reactive oxygen species (ROS) and other reactive compounds, such as aldehydes [15]. At the same time, CS causes a decrease of endogenous antioxidants efficiency contributing to ascertain the process called oxidative stress, which often results in indiscriminate damage to cells and tissues [16].

Indeed, in patients with COPD many studies have showed increased levels of several markers for oxidative stress, such as 8-hydroxy-deoxyguanosine (8-OHdG), a well-known index of oxidative DNA damage [17], and indices of lipid peroxidation, including 4-hydroxynonenal (4-HNE) and malondialdehyde [18, 19]. Of note, the increase of these oxidative markers was positively correlated with disease severity and negatively associated with lung function [18, 20]. In addition, deficiency in antioxidants was showed in smokers and COPD patients besides the ROS increased levels. The expression and activity of antioxidant enzymes such as Manganese Superoxide Dismutase (MnSOD), Catalase (Cat), and Glutathione-S-Transferase (GST) have been found deranged during COPD [21, 22], and a positive correlation between the deficiency of antioxidant status and the severity of COPD exacerbations was reported [23].

It is now clear that a global alteration of cellular redox homeostasis is detectable in smokers and COPD patients. In this context, it is crucial to consider that several molecular factors modulating the expression and activity of the antioxidant enzymes are impaired during COPD. For instance, Hwang et al. found that the levels of FoxO3, a member of the Forkhead box class O (FoxO) regulating the expression of MnSOD, Cat, and other antioxidants in response to oxidative stress, were significantly decreased in lungs of smokers and COPD patients, as well as in lungs of mice exposed to CS. The authors demonstrated that the absence of FoxO3 reduced the protein expression of MnSOD and Cat in response to CS, resulting in the oxidative stress accumulation in the lung [24]. Apart from FoxO3, other redox-sensitive factors, such as nuclear factor erythroid 2-related factor 2 (NRF2), nuclear factor *κ*B (NF-*κ*B), and activator protein 1 (AP-1), are deranged in COPD patients resulting in deficiency of antioxidant defense system and consequentially in a status of persistent oxidative stress [25–27].

## 3. Inflammation and COPD

Since the 1990s, ROS accumulation and/or antioxidant system inefficiency have been identified as crucial events prompting inflammatory response [28]. A buildup of oxidants induces a further production of ROS from inflammatory cells infiltrating the lung, favoring a vicious circle that leads to uncontrolled inflammatory response [29, 30]. Increased levels of ROS trigger the activation of the above-mentioned redox-sensitive transcriptional factors, which, in turn, lead to increased production of proinflammatory cytokines including interleukin-1 (IL-1), interleukin-6 (IL-6), and Tumor Necrosis Factor *α* (TNF*α*) [31]. Also for oxidative stress, the level of airway inflammation is increased during COPD exacerbations and the maintenance of inflammation seems to be a phenomenon oxidant mediated, suggesting that oxidative stress and chronic inflammation in COPD may be strongly linked [23]. Due to concomitant action played by ROS, antioxidant enzymes, and cytokines in both development and progression of COPD, a number of antioxidant agents, exhibiting both antioxidant and anti-inflammatory effects, had been proposed for COPD treatment and management [16, 32]. Nowadays, it seems that the best therapeutic approach should be to inhibit the accumulation of oxidative stress and the subsequent inflammatory response and also to counteract the excess of ROS generation working on redox-sensitive signal transduction pathways. The stimulatory attitude of ROS to trigger inflammation has been also linked to epigenetic processes, including methylation, phosphorylation, and acetylation [33]. In this regard, the balance between acetylation and deacetylation is crucial not only for gene transcription control, but also for a direct regulation of the nuclear activity of transcription factors driving chronic inflammation [33, 34].

Among the great number of inflammatory mediators contributing to delineate COPD phenotypes, Matrix Metalloproteinase-9 (MMP-9) has deserved particular attention from the scientific community. It was found increased in COPD patients when compared to control subjects with normal lung function [35].

Of note, there is a link among MMP-9 levels and oxidant/antioxidant and acetylation/deacetylation balance with implications in response to treatments for respiratory diseases [36], particularly for glucocorticoid therapy management in COPD [37, 38].

## 4. Aging/Senescence and COPD

Evidence is accumulating in favor of the concept that oxidative stress and inflammation, which are interdependent processes, are also strictly correlated to cellular senescence and aging. With advancing age, humans are continuously exposed to endogenous and environment antigens, which lead to a remodeling of both innate and acquired immune systems with a consequent establishment of chronic inflammatory state, referred to as inflammaging [39]. This process is characterized by activation of several signaling molecules, such as NF-*κ*B, Forkhead box O, and Klotho, and raised levels of proinflammatory cytokines [40].

During their life, the cells progressively impair the ability to defend themselves from stress stimuli with consequent collection of oxidative damage in all cellular constituents [41, 42].

The cell signaling pathways implicated in inflammaging are also involved in several age-associated diseases, including COPD, sharing an inflammatory basis and high levels of oxidative stress [43–45].

Importantly, glucocorticoid sensitivity is reduced during aging as well as in COPD patients. For example, changes in cellular responses to glucocorticoid [46] and decline in the expression of glucocorticoid receptors [47] were reported in both elderly and COPD patients.

According to a well-known aging theory, the telomeres shortening is a potent inducer of cellular senescence, and the critical involved components are represented by the telomerase reverse transcriptase (TERT) and the telomerase RNA (TR or TERC) [48].

To corroborate the aging hypothesis for COPD, some studies in families with idiopathic pulmonary fibrosis have indicated that telomerase mutations play a critical role in the genetics of lung disease. Alder et al. [49] demonstrated that short telomere length is a genetic determinant of emphysema in mice and may contribute to the susceptibility to CS-induced lung disease with age in humans. The authors [49] showed that the telomere-associated emphysema susceptibility did not depend on a telomere defect in bone marrow-derived cells and short telomeres caused emphysema susceptibility independent of inflammatory cell genotype, indicating that although the inflammatory response after CS is striking, the recruitment of macrophages per se was not sufficient to induce emphysema in this model. In addition, in epithelial cells, DNA damage caused by CS is additive to telomere dysfunction with short telomere mice carrying the greatest burden, suggesting that although short telomere length alone does not cause disease phenotypes, the combined telomere and CS-induced damage together overcome a threshold and manifest as emphysema [49].

Recently, Stanley et al. [50] reported that germline mutations in telomerase are a risk factor for severe emphysema in smokers by the evidence, in two independent humans cohorts, that 1% of cases carried deleterious mutations in TERT. The emphysema-associated TERT variants compromised telomerase catalytic activity, and mutation carriers had abnormally short telomeres [50].

Another evidence sustains the involvement of other aging-related molecules in the COPD process, as in the case of Nrf2 that plays a critical defensive role against oxidative and cytotoxic stress [51]. Nrf2 production appears to decline with age, while ROS amount increases [52]. However, deficiencies in antioxidant and cytoprotective response due to an impaired Nrf2 function have been also linked to major disorders including cancer and neurodegenerative as well as cardiovascular and pulmonary diseases [53, 54].

COPD is an example of a disease of accelerated aging where Nrf2 is decreased [55]. Defective Nrf2 can explain the excessive oxidative stress present in the lungs that causes damage and inflammation [25, 55]. Recently it has been demonstrated that pharmacological activation of Nrf2 by sulforaphane, a potent activator of Nrf2, leads to restoration of corticosteroid responsiveness in alveolar macrophages from COPD patients [56].

Another molecule is the mammalian TOR (mTOR), also acknowledged as “mechanistic TOR,” involved in the switch between catabolic and anabolic states to tolerate variable nutrient availability [57]. The mTOR signalling is involved in several age-related diseases, including cancer, diabetes, and neurodegeneration with a consequent important role in aging and lifespan control [58].

The inhibition of TOR signalling, either genetically or pharmacologically, can induce life extension in many species [59–61], including mammals [62]. Moreover, some evidence indicates that mTOR directly regulates components of the autophagic machinery, a survival mechanism acting in response to cellular stress [63]. Increased autophagy has been demonstrated in lung tissue from COPD patients but not in lungs from patients with other pulmonary diseases [64].

Starting by the evidence that particulate matter (PM2.5) air pollutants have been shown to exacerbate a variety of pulmonary disorders, including COPD, Wang et al. demonstrated that phosphorylation of AMPK and dephosphorylation of mTOR were observed following PM2.5 treatment. In addition, they showed that PM2.5-induced autophagy conferred a prosurvival role in host defense. The authors suggested that, in response to PM2.5-induced increase in cellular ROS [65], a self-protective mechanism was elicited by inducing autophagy via the AMPK pathway.

Blocking of mTOR has been proposed as a therapeutic option to treat age-related disorders, including respiratory diseases, but at present it is not clear whether this is feasible in emphysema [44].

## 5. SIRT1 and COPD

Up to date, several studies suggest an important role of enzymes' family, termed sirtuins, in the pathogenesis of COPD [10, 11]. Sirtuins (SIRT1–SIRT7) are NAD^+^ dependent deacetylases that control a wide number of processes implicated in the regulation of homeostasis [66]. Although the original identification as a prolonging lifespan is now under debate, there is no doubt that SIRT1 (the best characterized member of sirtuins in mammals) has a key role in governing cellular stress management and, consequently, in healthy lifespan [67].

SIRT1 is implicated in aging and senescence [42, 68–70], age-associated conditions [71], including diabetes [72], neurodegenerative syndromes [73], and cardiovascular diseases [74, 75].

Through deacetylation of many transcriptional factors, SIRT1 modulates oxidative stress response, endothelial dysfunction, and inflammation, all key events acting in premature cellular senescence and aging [76] and also implicated in COPD onset and progression [77, 78].

The idea that SIRT1 might be implicated in COPD development and progression is relatively recent; nevertheless, very interesting investigations have been already performed in this field.

Rajendrasozhan et al. for the first time demonstrated that Sirt1 is decreased in lungs of smokers and COPD patients and that such reduced levels were inversely correlated to increased posttranslational modifications of the protein by increasing amount of reactive aldehyde 4-hydroxy-2-nonenol and 3-nitrotyrosine, markers of oxidative and nitrosative stress, respectively. Moreover, in monocyte-macrophage (MonoMac6) cell line exposed to cigarette smoke extract (CSE), increasing concentrations of CSE corresponded to decreasing levels of both SIRT1 mRNA and protein, and to increasing release from the cells of interleukin-8 (IL-8) [79].

Importantly, these authors also demonstrated that decreased levels of SIRT1 expression were associated with increased activation of RelA/p65 NF-*κ*B, the master regulator of inflammation, already reported as factor directly controlled by SIRT1 [80].

Notably, both oxidative and nitrosative stress induced by CSE are strongly correlated to the release of proinflammatory molecules, and oxidant/antioxidant imbalance has been associated with the staging of COPD and corticosteroid resistance [81, 82].

It was also showed that a reduction of SIRT1 induced by oxidative stress causes an increase of MMP9 and, conversely, increased SIRT1 activity counteracts such elevation [83].

MMP9 is involved in the breakage of extracellular matrix and, actually, it is greatly increased in patients with COPD [84] and also in subjects affected by other inflammatory diseases, including asthma and lung cancer [85], as well as in the elderly [86].

Nakamaru et al. recorded a progressive decline in SIRT1 expression and activity in the lung of COPD patients, while transcripts levels of MMP9 and IL-8 were increased with disease severity [83].

Investigations performed in macrophage/monocytes cell lines exposed to increasing doses of H_2_O_2_ have revealed that oxidative stress affects firstly the activity and then the expression of SIRT1, which, in turn, controls the mRNA levels of MMP9 [83].

This oxidant-mediated mechanism was also found* in vitro* where the expression of other inflammatory molecules, such as RelA/p65 NF-*κ*B and IL-8 [79], and of the well-known prosenescent markers p21 and p16 was regulated by SIRT1 [87]. These findings corroborate the existence of a crosstalk between ROS accumulation and SIRT1 and suggest that this deacetylase could represent a connecting link between oxidative stress and inflammation [67].

Recently, it was shown that SIRT1 expression progressively diminished in the lungs of patients with mild and severe COPD when compared with “healthy” smokers, and it occurs for the expression of deacetylases of class I HDAC2 and for other molecules, such as T-box family transcription factors [88]. Interestingly, while antisenescence factors like SIRT1 decreased, the expression levels of prosenescent factors, such as cyclin-dependent kinase inhibitor 2A and caveolin-1, increased in a COPD staging-dependent manner [89]. Isajevs et al. showed that the expression of SIRT1 decreased in both large and small airways of patients with COPD when compared with nonsmokers and smokers controls. Of note, SIRT1 was much more suppressed in small airways of patients than in those of smokers with normal lung function, suggesting that the decrement in SIRT1 expression might be associated with an increase of inflammatory cells in small airways of the patients [90]. Recently, Yao et al. in two consecutive studies have well illustrated the interconnection existing among oxidative stress, inflammation, and cellular senescence. In a first study, the protein level of SIRT1 was decreased in the lung of heterozygous knockout (Sirt1+/−) mice exposed to CS and elastase and in the lung of patients with COPD/emphysema. Sirt1 overexpression attenuated the increased levels of the senescent markers p21, p16, and p53 in the lung of Sirt1+/− mice and protected against increase of lung senescence (assessed by SA-*β*-gal activity) induced by CS or elastase [87]. In the second investigation, both SIRT1 genetic overexpression and pharmacological activation demonstrated to contribute in combatting lung emphysema by regulating the imbalance between oxidants and antioxidants induced by CS exposure. Notably, SIRT1720, a specific SIRT1 activator, increased the activity of SIRT1 in mice lung favoring a recovery of the expression of several antioxidant genes, previously repressed by CS exposure, via Foxo3a, a well-known SIRT1 target already implicated in aging and cellular senescence [91, 92].

Finally, together with previous investigations [92, 93], the studies by Yao et al. [87] showed that both SIRT1 expression and SIRT1 activity were reduced in an age-dependent manner. The SIRT1-deficient mice developed an emphysematous phenotype after 1 year of age although the reduction of SIRT1 levels was started at 6–8 months of age in the lung of these rodents [87], suggesting one more time a role of SIRT1 in mediating the aging-associated features of COPD.

More recently, some researchers have pointed the attention to another member of sirtuins, SIRT6. Loss of SIRT6 increased the expression of NF-*κ*B, whereas overexpression of SIRT6 was associated with decreased NF-*κ*B transcriptional activity in endothelial cells [94]. The expression of SIRT6 was found decreased in lung homogenates from COPD patients and suppressed in Human Bronchial Epithelial Cells (HBECs) exposed to CSE. In addition, SIRT6 regulated CSE-induced cell senescence in HBECs [95].

**Figure 1:**
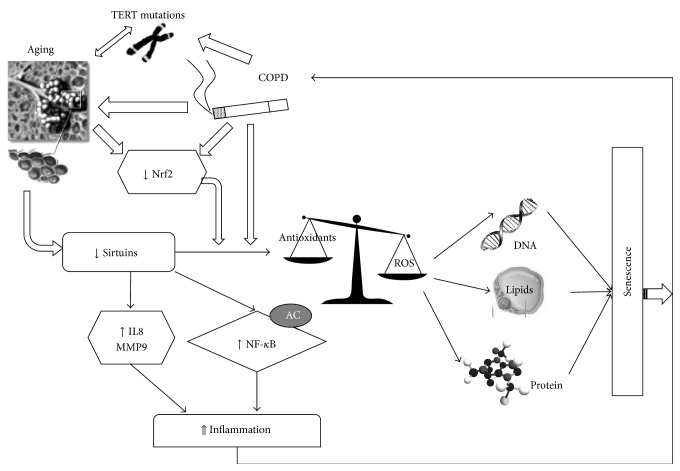
“The aging theory of COPD.” Several molecules involved in aging are also implicated in COPD pathogenesis: mutations in TERT are responsible for telomeres shortening; decreased Nrf2 can explain the excessive oxidative stress that causes damage and inflammation during both aging and COPD. Recent studies have suggested a crucial role of SIRT1 in COPD. Aging of lung is characterized by reduction in SIRT1 activity and expression. Decreased levels of SIRT1 lead to increased acetylation of NF-*κ*B and are responsible for a persistent activation of proinflammatory molecules, such as IL8 and MMP9, and also for raised levels of senescence through accumulation of oxidants at cellular constituents (DNA, lipid, and protein). At the same time, COPD and smoking habit are associated with decreased SIRT1 activity and consequently increased inflammation and senescence. Finally, a vicious circle occurs with inflammation and senescence that further deteriorate COPD and aging conditions.

It is conceivable that SIRT6 could be representing another promising therapeutic target for COPD because of its involvement in the determination of aging phenotypes [95–97]. The results about SIRT6, together with those on SIRT1, contribute to sustain the “aging theory for COPD” (Figure 1).

## 6. Conclusion 

There is still a considerable lack of knowledge about the molecular mechanisms responsible for COPD onset and progression. As a consequence, current pharmacological treatments are still inadequate to reduce the disease progression and mortality. To elucidate molecular pathways underlined COPD pathogenesis could be helpful to identify valid biomarkers and to develop new pharmacological therapies allowing a better clinical management of the disease.

Increasing evidence indicates SIRT1 as a connecting link among oxidative stress, inflammation, and aging/senescence, all processes characterizing COPD phenotypes, especially COPD/emphysema.

These findings contributed to stressing the hypothesis that COPD could be considered a disease of accelerated aging and underline the potential of SIRT1 as valid therapeutic target to treat respiratory disorders sharing chronic inflammation.
